# Cytotoxic endophyte from *Camellia oleifera* Abel - *Penicillium chermesinum* NR121310

**DOI:** 10.1080/21501203.2019.1705928

**Published:** 2019-12-18

**Authors:** Surasak Prachya, Chakree Wattanasiri, Acharavadee Pansanit

**Affiliations:** a Chulabhorn Research Institute, Bangkok, Thailand; b School of Integrative Medicine, Mae Fah Luang University, Chiang Rai, Thailand; c School of Science, Mae Fah Luang University, Chiang Rai, Thailand

**Keywords:** Endophytes, *Penicillium chermesinum*, *Camellia oleifera* Abel, antioxidant, cytotoxic

## Abstract

Ten unknown endophytes were isolated from several parts of *Camellia oleifera* Abel and their biological activities were studied. One endophyte, Tea2-L1, showed the highest inhibition in various antioxidant capacity assay and cytotoxicity assays. The phylogenetic study suggested that Tea2-L1 endophyte is identified as *Penicillium chermesinum* NR121310 species. This shows the importance of the *Penicillium* species as a potential drug source.

## Introduction

Endophytes are organisms inhabiting plant organs at some point in their life. They can colonise internal plant tissues without harming the host. The endophytes may have established this relationship with their host during their evolution (Hyde and Soytong 2008). Endophytes produce various types of secondary metabolites such as phenols, benzopyroanone, terpenoids, palmarumycins, furandiones, and dimeric anthrone (Schulz et al. 2002). Taxol is one of the famous anticancer drug that is currently used around the world. It was first discovered from the Yew tree (*Taxus brevifolia*) and also found in the endophytes of *T. brevifolia* (Stierle et al. 1993). Not only anticancer agents are found in endophytes but endophytes are also a major source for new and useful metabolites. There are reports in the discovery of these useful metabolites which are produced from endophytes, such as the species of *Aspergillus, Fusarium, Acremonium* and *Penicillium* (Dreyfuss and Chapela 1994; Hyde and Soytong 2008).

In this study, we investigated the endophytes isolated from *Camellia oleifera* Abel, which demonstrated potent biological activities. *C.oleifera* Abel or tea oil belongs to the Camellia family. It is widely distributed in the southern part of China and also found in the northern region throughout South East Asia. Previous studies on *C.oleifera* Abel led to the isolation of chemical constituents from several part of this plant. The studies on the chemical compounds in the seed of *C.oleifera* Abel revealed that it contains many antioxidants and antimicrobial agents. Among these compounds 82–84% are unsaturated fatty acids (Ma et al. 2011), while other reported compounds are sesamin, saponin and 2,5-bis-benzo[1,3]dioxol −5-yl-tetrahydro-furo[3,4-d][1,3]-dioxine (Lee and Yen 2006). Moreover, the saponin derivatives found in seed and seed cake of *C.oleifera* Abel showed interesting biological activities such as antibacterial, antifungal, and anticancer activities (Zhang et al. 2012; Zhang et al. 2014; Zhu et al., 2018). From previous studies, *C.oleifera* Abel is a good source of bioactive agents. However, they are not many studies on the endophytes found on *C.oleifera* Abel.

From this study, we found several endophytes in different parts of *C.oleifera* Abel. However, one particular endophyte (Tea2-L1) showed to be a potent extract in terms of antioxidant capacity and cytotoxic activities on cancer cell lines, which was later identified as *Penicillium* sp. The *Penicillium* species belongs to ascomycetous genus which is important in the environment. Some member of this genus produces well known and important chemical agents, such as penicillin. This important antimicrobial agent, was discovered in a *Penicillium sp*. by Alexander Fleming in 1928 (Sykes 2001). The results from this work would provide valuable information for the discovery of new medicinal agents in the future.

## Results and discussion

Ten endophytes were isolated from different parts of *C.oleifera* Abel. All of the cultures were cultivated in 500 mL of PDB. The amount of crude extracts used are in the range of 12.0–175.2 mg. The crudes were studied on antioxidant capacity and cytotoxic activity as shown in Tables 1 and 2, respectively. From the antioxidant capacity test in Table 1, most of the crude extract show antioxidant capacity towards inhibiting the production of radicals, as seen in the DPPH and XXO assay. On the other hand, the crude mycelia extract of Tea2-L1 shows high antioxidation capacity in the DPPH assay along with XXO, IXO and also LOX assays. This can imply that the active compounds in the crude mycelia extract of Tea2-L1 can inhibit not only the free radical formation but also inhibit the enzyme activity as well, as seen in IXO and LOX assays. For the chemoprevention assays which are the inhibition of aromatase enzyme (CYP19) and the inhibition of 12-O-tetra-decanoylphorbol-13-acetate (TPA)-induced superoxide anion radical generation in differentiated HL-60 cell lines (HL-60 assay), none of the crude extracts showed any activities (result not shown).

**Table 1.** Percentage inhibition of an antioxidant activity of crude extract of endophytes from *C.oleifera* Abel (mean, *n* = 3)

No.	Crude extracts	Amount of crudeextracts (mg)	DPPH	XXO	IXO	LOX
1	Tea2-T1-CBE	16.8	-	A (64%)	-	-
2	Tea2-T1-CME	27.9	-	-	-	-
3	Tea2-T2- CBE	175.2	A (53%)	A (96%)	-	-
4	Tea2-T2-CME	98.1	-	A (97%)	-	-
5	Tea2-T3- CBE	51.1	A (54%)	A (83%)	-	-
6	Tea2-T3-CME	52.6	-	A (53%)	-	-
7	Tea2-T4-CBE	12.0	-	A (76%)	-	-
8	Tea2-T4-CME	34.2	-	-	-	-
9	Tea2-T6- CBE	53.7	A (74%)	A (63%)	-	-
10	Tea2-T6-CME	134.7	-	-	-	-
11	Tea2-T7-CBE	37.0	A (74%)	-	A (83%)	-
12	Tea2-T7-CME	79.3	A (51%)	A (65%)	-	-
13	Tea2-L1- CBE	91.7	A (91%)	A (97%)	-	-
14	Tea2- L1-CME	93.0	A (80%)	A (88%)	A (58%)	A (72%)
15	Tea2- L2-CBE	28.6	-	A (87%)	-	-
16	Tea2- L2-CME	139.5	-	-	-	-
17	Tea2- L3-CBE	47.9	-	A (85%)	-	-
18	Tea2- L3-CME	77.2	-	A (53%)	-	-
19	Tea2- Fl1-CBE	37.7	A (64%)	A (79%)	-	-
20	Tea2- Fl1-CME	83.4	inactive	**A (75%)**	inactive	inactive
21	Ascorbic acid (IC_50_, µM)	-	25.6 ± 2.1	ND	ND	ND
22	Allopurinol (IC_50_, µM)	-	ND	ND	4.5 ± 0.3	ND
23	Nordihydroguaiaretic acid (IC_50_, µM)	-	ND	ND	ND	4.9 ± 0.3

All of the crudes are not active on aromatase inhibition and HL-60 assays (%inhibition less than 50%) which do not show the data in the table. A = active. ND = not determined. The percentage inhibition of the crudes that less than 50% are reported as no activity (-).

**Table 2.** Cytotoxic activity of crude extracts of endophytes from *C.oleifera* Abel (mean, *n* = 3)

No.	Crude extracts	Moult-3	HuCCA-1	No.	Crude extracts	Moult-3	HuCCA-1
1	Tea2-T1-CBE	-	-	12	Tea2-T7-CME	-	-
2	Tea2-T1-CME	-	-	13	Tea2-L1- CBE	-	-
3	Tea2-T2- CBE	**A (70%)**	-	14	Tea2- L1-CME	**A (77%)**	**A (67%)**
4	Tea2-T2-CME	-	-	15	Tea2- L2-CBE	-	-
5	Tea2-T3- CBE	-	-	16	Tea2- L2-CME	-	-
6	Tea2-T3-CME	-	-	17	Tea2- L3-CBE	-	-
7	Tea2-T4-CBE	-	-	18	Tea2- L3-CME	**A (62%)**	-
8	Tea2-T4-CME	-	-	19	Tea2- Fl1-CBE	-	-
9	Tea2-T6- CBE	-	-	20	Tea2- Fl1-CME	-	-
10	Tea2-T6-CME	-	-	21	Doxorubicin (IC_50_, µg/mL)	0.010 ± 0.003	0.5 ± 0.028
11	Tea2-T7-CBE	-	-	22	Etoposide (IC_50_, µg/mL)	0.020 ± 0.003	ND

The cytotoxic activity was tested against the following cell lines using the screening concentration at 10 µg/mL: HepG2 (human hepatocellular liver carcinoma), HuCCA-1 (human lung cholangiocarcinoma), A549 (human lung carcinoma) and Moult-3 (acute lymphoblastic leukaemia) cell lines. However, all of the crude extracts could not inhibit HepG2, and A549 cell lines (%inhibition less than 50%). A = active. ND = not determined. The percentage inhibition of the crudes that less than 50% are reported as no activity (-).

For the cytotoxicity properties are shown in Table 2. The crude extracts did not inhibit the HepG2 (human hepatocellular liver carcinoma) and A549 (human lung carcinoma) cell lines (result not shown). This can suggest that the crude extracts are not potential candidates of anticancer agents for liver carcinoma and lung carcinoma. However, crude mycelia extract of Tea2-L1 endophyte (Tea2-L1-CME) showed higher cytotoxic activities compared to other crude extracts (Table 1). Most of the crude extract are not active against the Moult-3 and HuCCA-1 cell lines. There were 3 crude extracts that can inhibit the Moult-3 cell line, which are Tea2-T2-CBE, Tea2-L1-CME and Tea2-L3-CME. Only Tea2-L1-CME can inhibit both Moult-3 and HuCCA-1 cell lines with high inhibition percentage at the testing concentration of 10 µg/mL. This result suggests that Tea2-L1 endophyte is an interesting lead of endophytic fungi from *C. oleifera* Abel. It can be used to as a platform for investigating promising antioxidant and anticancer agents in the future or might even be a source of bioactive compounds with these properties.

**Figure 1.**
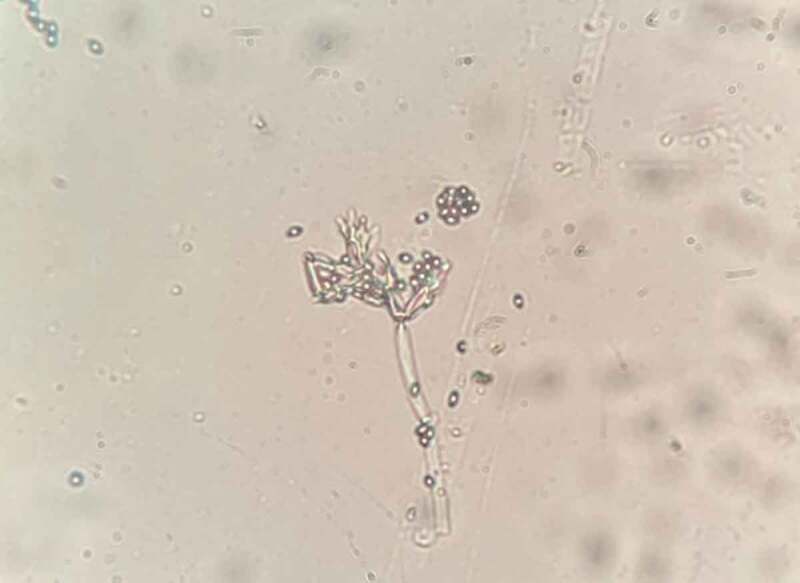
The micro-morphology of the hypha and spore of *Penicillium sp*. Tea2-L1

**Figure 2.**
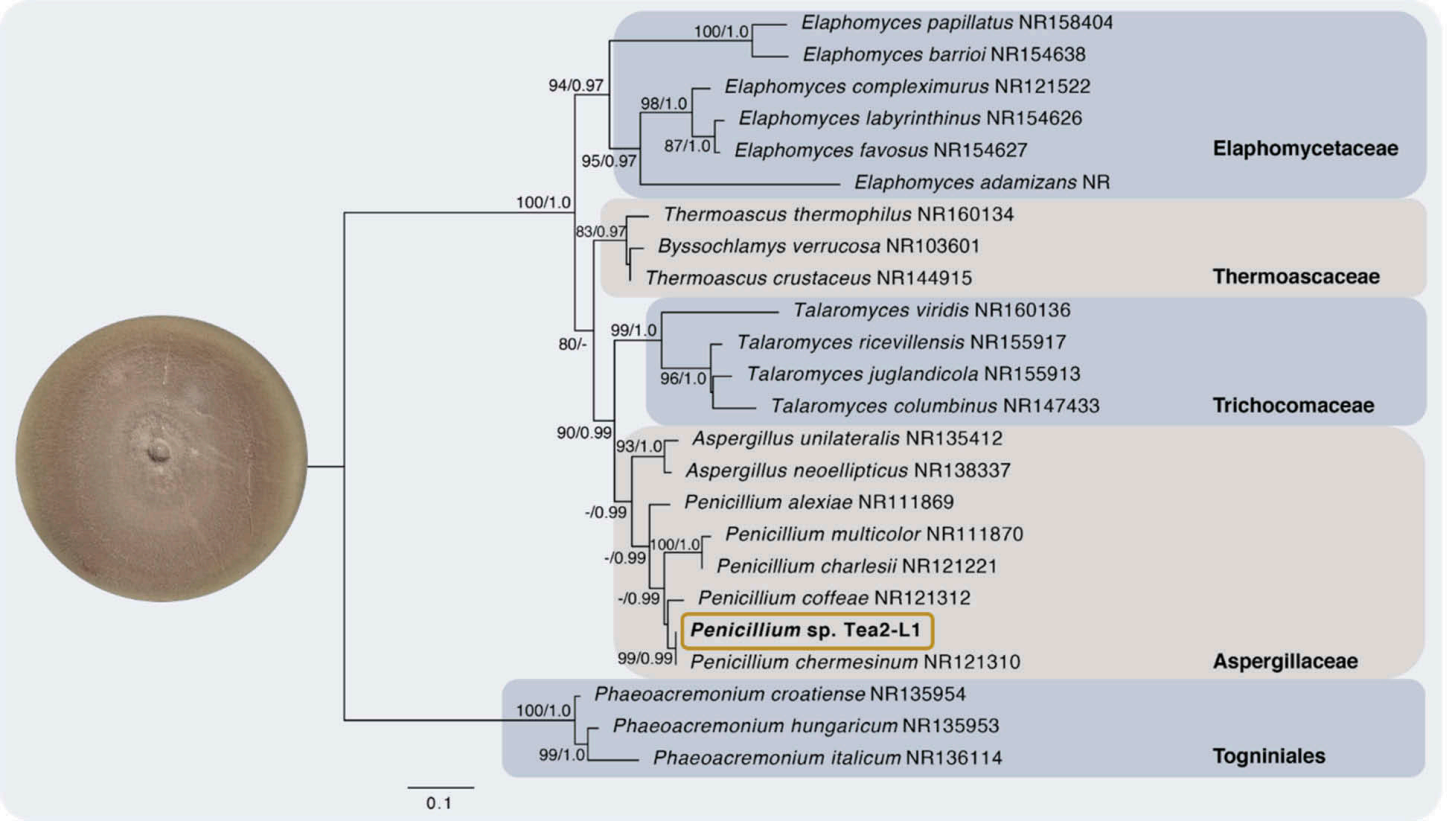
Phylogenetic tree of internal transcribed spacers inferred from 51 taxa and 830 sites under the GTR model of nucleotide substitution of the newly sequenced endophytic fungus *Penicillium* sp. Tea2-L1 is in bold with the box. Bootstrap support values for maximum likelihood greater than 70% and Bayesian inference posterior probabilities greater than 0.99 are shown

From the results, the Tea2-L1 endophyte was determined. The phylogenetics analysis has been demonstrated repeatedly. Molecular and phylogenetic methods were employed to further confirm the identification of the Tea2-L1 endophyte from *C.oleifera* Abel. The tree topologies indicated the similarity between the maximum likelihood and Bayesian inference methods. The newly sequenced Tea2-L1 endophyte from *C.oleifera* Abel showed a grouping up with Aspergillaceae within the genus *Penicillium* clade with maximum support (99BS/0.99PB). This indicates that it does indeed belong to the *Penicillium* genus. This data suggested that the *Penicillium* sp. Tea2-L1 is the *Penicillium chermesinum* NR121310 species. The micro-morphology of hypha and spore and phylogenetic tree of Tea2-L1 is shown in Figures 1 and 2, respectively. This result also supports the biological activities of the *Penicillium* sp. Tea2-L1, which showed to have both high antioxidant capacity and cytotoxic activity. Previous reports, showed that *Penicillium* genus has many important biological activities (Hyde and Soytong 2008). Moreover, Kittakoop’s group discovered a new polyketide (2-chloro-3,4,7-trihydroxy-9-methoxy-1-methyl-6*H*-benzo[c]chromen-6-one) from the *Penicillium chermesinum* which has shown to be cytotoxic against four cancer cell lines, which are HuCCA-1, HepG2, A549 and Moult-3 cell lines (Darsih et al. 2017). This data supports that the *Penicillium* sp. Tea2-L1 can be used as a lead source for cancer drug discovery.

## Conclusion

The *Penicillium* sp. Tea2-L1 is an endophytic fungus isolated from *C. oleifera* Abel which has good biological activities in antioxidant capacity and cytotoxic activities. These results will be useful for the development of antioxidant and cytotoxic agents in the future.

## Materials and methods

### Plant materials

Fruits, leaves, twigs, and Bark of *C. oleifera* Abel were collected in May 2018 from the Tea Oil and Plant Oils Development Centre in Mae Sai, Chiang Rai, Thailand.

### Isolation of endophytic fungi

*C.oleifera* Abel samples were cleaned under running tap water and then air-dried. The cleaned stems (bark, leaves, twigs and fruits) were cut into small pieces of ca.5 cm long leaves before surface sterilisation. The fragments were sterilised by soaking in 70% ethanol for 1 min, 5% sodium hypochlorite solution for 5 min, and sterile distilled water for 1 min two times. After the sterilisation process, the fragments were air-dried in a laminar flow chamber. The surface-sterilised leaves and stems were cut into small pieces using a sterile blade and then transferred to sterile water agar plates for incubation at 30°C. The hyphal tip of endophytic fungi growing out from the plant tissue was cut by a sterile pasture pipette and transferred to a sterile potato dextrose agar (PDA) plate. After incubation at 30°C for 7–14 days, pure culture was determined from colony morphology (Gerhäuser et al. 2003; Hata and Sone 2008; Pansanit et al. 2018).

### Genomic DNA extraction of Penicillium chermesinum and PCR

*Penicillium chermesinum* was selected to complement the morphological identification due to its promising antioxidant and chemoprevention activities, which were notably higher than other endophytic fungal extracts. The aerial mycelium of strain Tea2-L1 was scraped from the PDA surface. This fungal biomass was further pulverised into a fine powder with a pestle and mortar. The genomic DNA of this strain was extracted using cetyltrimethylammonium bromide (CTAB) according to manufacturer’s specifications. CTAB has previously been used to successfully extract nucleic acids from endophytes (Ye et al. 2012). To supplement the database with molecular information of the new isolate the barcode fragment ITS1-5.8S-ITS2 sequence was amplified using the universal primers, ITS1 (5ʹ-TCC GTA GGT GAA CCT GCG G-3ʹ) and ITS4 (5ʹ-TCC TCC GCT TAT TGA TAT GC-3ʹ). The PCR conditions were: 95°C for 5 min, followed by 40 cycles of 95°C for 50 s, 52°C for 50 s, 72°C for 50 s and final extension at 72°C for 10 min on a PeqSTAR 2x thermal cycler (Peqlab, Germany). PCR products were checked on 1% agarose gels stained with ethidium bromide under UV light and purified using NucleoSpin® Gel and PCR Clean-up Kit (Macherey-Nagel, Germany). The purified PCR products were directly sequenced in both directions at the 1^ST^ Base Company (Malaysia) using the same PCR primers mentioned above. The acquired gene sequence was submitted to the NCBI Genbank database under an accession number as MH861332.1.

### Phylogenetic analysis

The newly acquired sequence was subjected to BLAST search against the National Centre for Biotechnology Information (NCBI) database to exclude bacterial contamination. Following this, we interrogated the nr database using blastn and assembled our dataset, which contained representative taxa spanning the Xylariomycetidae. Sequences were aligned using MAFFT (default parameters) and trimmed with trimAl, automated option. After trimming, 830 sites remained in the alignment. A maximum likelihood phylogenetic tree was constructed using RAxML-HPC v.8 and the general time reversible model of nucleotide substitution. Bootstrap values were obtained from 1000 replicates. In order to further confirm our phylogenetic hypothesis, a Bayesian inference analysis was also performed, whereby four Monte Carlo Markov chains were run for 1,000,000 generations. Convergence was declared when all RI parameters converged towards zero.

### Fermentation and extraction

The endophyte cultures were cultured on PDA at 30°C for 7 days and cut in small pieces (6 mm of diameter). The pieces of culture PDA were transferred into a 1000mL Erlenmeyer flask containing 150 mL of potato dextrose broth (PDB) and incubated at 30°C for 30 days. The fungal cultures were separated in broth and mycelia parts by simple filtration. The broth layer was extracted three times with ethyl acetate and concentrated by a vacuum rotary evaporator at reduced pressure at 40°C, resulting in the crude broth extracts (CBE). The mycelia layer was sequentially macerated in methanol at room temperature for 2 days and followed by dichloromethane. The mycelia methanol and dichloromethane extracts were combined and further extracted three times with ethyl acetate (Pansanit and Pripdeevech 2018) to provide crude mycelia extracts (CME).

### Cytotoxic assay

The test against HepG2 (human hepatocellular liver carcinoma), HuCCA-1 (human lung cholangiocarcinoma) and A549 (human lung carcinoma) cancer cell lines were performed by using MTT assay (Gunasekaran et al. 2017), while Moult-3 (acute lymphoblastic leukaemia) and HL-60 (human promyelocytic leukaemia) cell lines were investigated by using XTT assay (Strobel et al. 1997). The reference drugs of the cytotoxic assays were doxorubicin, doxorubicin hydrochloride and etoposide (IC_50_ values are shown in Table 1).

### Scavenging 2,2-diphenyl-1-picrylhydrazyl (DPPH) free radicals

The activity on radical scavenging was evaluated using DPPH assay. This method was carried out as described by (Gerhäuser et al. 2003). The solvent was used as a blank (presenting at 0% radical scavenging) and 250 µM ascorbic acid as the reference compound, which has 100% radical scavenging capacity. The half maximal scavenging concentration or IC_50_ value of ascorbic acid is at 25.6 ± 2.1 µM.

### Inhibition of superoxide anion radical formation by xanthine/xanthine oxidase (XXO assay)

XXO is inhibition of superoxide anion radical formation by xanthine/xanthine oxidase and HL-60 is the inhibition of 12-O-tetra-decanoylphorbol-13-acetate-induced superoxide anion radical generation in differentiated HL-60 cell lines. The XXO assay was performed following the described method by (Gerhäuser et al. 2003). Superoxide dismutase enzymes was used as a negative control, which has 100% radical scavenging capacity. Allopurinol was used as the reference compound which showed the inhibition on xanthine oxidase (IXO) at IC_50_ value of 4.5 ± 0.3 µM. Inhibition of superoxide anion radical formation was measured only when the tested compounds did not inhibit xanthine oxidase.

### Inhibition of 12-O-tetra-decanoylphorbol-13-acetate (tpa)-induced superoxide anion radical generation in differentiated HL-60 cell lines (HL-60 assay)

This assay was carried out according to the method described by (Gerhäuser et al. 2003). TPA-induced superoxide anion radical formation in differentiated HL-60 (human promyelocytic leukaemia) cells was detected by photometric determination of Cytochrome-C reduction. The positive control was 60 U/mL of superoxide dismutase, which has 100% radical scavenging capacity. This assay considered only the sample that has more than 50% cell viability for the calculation of scavenging potential.

### Inhibition of aromatase (CYP19)

Aromatase inhibition assay was carried out according to the method described by (Stresser et al. 2000). Letrozole was used as a positive control with IC_50_ value of 1.3 ± 0.0 nM.
